# The AFFIRM Framework for gender-affirming care: qualitative findings from the Transgender and Gender Diverse Health Equity Study

**DOI:** 10.1186/s12889-024-21261-7

**Published:** 2025-02-06

**Authors:** Meg Quint, Schuyler Bailar, Alexis Miranda, Shalender Bhasin, Devin O’Brien-Coon, Sari L. Reisner

**Affiliations:** 1 Stanford School of Medicine, Stanford, CA 94305 USA; 2 Department of Epidemiology, University of Michigan, Ann Arbor, MI 48109 USA; 3 Division of Endocrinology, Diabetes and Hypertension, Brigham and Women’s Hospital, Boston, MA 02115 USA; 4 Department of Epidemiology, Harvard School of Public Health, Boston, MA 02115 USA; 5 Research Program in Men’s Health: Aging and Metabolism, Claude D. Pepper Older Americans Independence Center, Brigham and Women’s Hospital, Boston, MA 02115 USA; 6 Division of Plastic Surgery, Brigham & Women’s Hospital, Harvard Medical School, Boston, MA 02115 USA; 7 Departments of Plastic Surgery and Biomedical Engineering, Johns Hopkins University School of Medicine, Baltimore, MD 21287 USA; 8 The Fenway Institute, Fenway Health, Boston, MA 02215 USA; 9 Department of Epidemiology, University of Michigan School of Public Health, 1415 Washington Heights, SPH 1-2649A, Ann Arbor, MI 48109 USA

**Keywords:** Transgender, LGBT health, Health equity, Healthcare quality

## Abstract

**Background:**

Transgender, nonbinary, and gender diverse (TGD) people experience stigma in healthcare settings impacting healthcare utilization, including avoidance of care due to anticipated discrimination. Gender-affirming care refers to care for medical gender affirmation, such as gender-affirming hormones and surgery, as well as general care that affirms and respects TGD patients. This study sought to explore the experiences of TGD adults to inform gender-affirming care delivery and develop an actionable framework for practice.

**Methods:**

Between May–October 2021, one-time individual in-depth interviews were conducted with 27 TGD adults receiving any healthcare in the greater Boston Massachusetts area to gather data about gender-affirming care. Interviews were semi-structured, explored prior and current experiences in healthcare and ideal gender-affirming care models, and conducted virtually via a secure Zoom platform. Analyses were conducted using immersion crystallization and reflexive thematic analysis; interview transcripts were double coded by two coders.

**Results:**

Participants had a mean age of 28.5, ranging 18–45 years, and were: 7 transgender men, 6 transgender women, 8 nonbinary, 3 genderqueer, 1 agender, and 2 gender not specified. Themes about gender-affirming care coalesced into the acronym AFFIRM: (1) Affirms in individual interactions: Participants called for affirmation of TGD identity, lived expertise, and competent TGD providers and staff. (2) Flexible and accessible: Participants expressed the need for gender-affirming care to be available beyond urban population-specific clinics, in a timely fashion without long wait lists, and in a community-centered manner such as offering non-traditional times and settings. (3) Fights systemic oppression: Participants emphasized the need for providers and health systems to eliminate gatekeeping practices for gender-affirming care and create care models that resist intersecting oppressive systems such as racism and cisgenderism. (4) Interacts with community: Patients desired intentional interaction with TGD community to holistically address health and unmet gender affirmation needs. (5) Retains patients in care: Patients shared the need to collaboratively identify and problem-solve obstacles to gender-affirming care with providers and healthcare systems to optimize TGD-specific retention strategies. (6) Multidisciplinary: Patients called for interdisciplinary teams with co-located services such as primary care and mental healthcare with letter-writing for surgical care, and incorporation of peer navigators to meet the broader social, health, and well-being needs of TGD people.

**Conclusions:**

Findings from this study and the AFFIRM Framework which emerged from TGD patient narratives can be applied to improve current care and set benchmarks for high-quality gender-affirming care delivery and practice.

**Supplementary Information:**

The online version contains supplementary material available at 10.1186/s12889-024-21261-7.

## Background

Transgender, nonbinary, and gender diverse (TGD) adults, an estimated 0.5% of the United States (U.S.) population [1], exhibit worse physical and mental health outcomes than the general population [2, 3], including in comorbid chronic health conditions [4], cardiovascular disease risk [5], HIV infection [6], suicidality [7], psychological distress [8, 9], preventive screening behaviors [10, 11], and mortality [12]. Clinical care is central to improving TGD population health. Medicine has begun to make progress in de-pathologizing TGD people, pushing for greater access to and competency in gender-affirming care [13, 14]. Yet, TGD individuals continue to experience discrimination and stigmatization in healthcare [15, 16], including care refusal and verbal abuse, and the consequences of mistreatment, such as healthcare avoidance mental health distress, and low care quality [17–22].

TGD individuals face unique care access barriers [22], including being less likely to be insured than cisgender adults [23]. Among the insured, TGD people are regularly denied coverage for gender-affirming surgery and routine preventive care [15]. TGD people avoid seeking necessary healthcare due to fear of experiencing transphobia [15]. Low quality care adversely affects engagement and retention in care. TGD patients who must teach providers about transgender people are four times more likely to delay needed care than those who have not had to educate providers [19]. TGD individuals report verbal harassment through misgendering, deadnaming (using name assigned at birth instead of chosen name), being asked invasive questions, and even experiencing physical assault [15, 16]. Structural oppression in healthcare systems, including erasure due to cisnormativity [24] and exclusionary institutional practices (e.g., absence of routine gender identity data collection), often shape TGD people’s individual clinical experiences and place the onus on individual TGD patients to remedy systemic challenges [25].

Gender-affirming care for TGD people refers to care specifically for medical gender affirmation, such as gender-affirming hormones and surgery, as well as to care generally that affirms and respects TGD patients [26, 27]. Clinical guidelines exist for gender-affirming care delivery, including those from the World Professional Association for Transgender Health Standards of Care Version 8 [14] and Endocrine Society Clinical Practice Guideline [28]. Prior work has articulated gender-affirming care models for TGD youth [29, 30]. The notion of community-driven gender-affirming care has been advanced in the Canadian context, describing the importance of intentional integration of community stakeholders to shape care and service provision for TGD adults [31]. Yet to our knowledge, no brief, accessible, and integrated framework exists for TGD adult care that has been derived from the experiences of TGD patients in the U.S. Improving care for the TGD population—both access to and quality of care—requires increasing the competency and ability of staff, providers, and medical systems. Integrated care frameworks have been previously articulated across a range of health issues, conditions, and populations to guide patient care provision [32–35], underscoring the utility and effectiveness of integrated frameworks to enhance care provision. Tangible and actionable TGD-specific frameworks are needed to guide medical professionals and healthcare systems in gender-affirming care.

This qualitative study sought to explore the healthcare experiences of TGD adults who received care in the Greater Boston Massachusetts Area and develop an actionable framework to guide gender-affirming care provision, centered in TGD patient experiences, for health systems and providers to evaluate and improve care delivery.

## Methods

### Participants and procedures

Between May 1, 2021 and October 1, 2021, qualitative data were collected as part of the Transgender and Gender Diverse Health Equity Study, a study conducted to explore the healthcare experiences of TGD individuals to inform enhancements in patient care. Individual, semi-structured, in-depth interviews were conducted virtually with 27 eligible TGD participants. Participants also completed a 10–15 min sociodemographic survey. Participants received a $50 gift card upon completion of study activities. Individuals were eligible for the study if they identified as TGD, were age 18 years or older, reported having a safe place to participate in a virtual interview, and reported receiving any healthcare in the Boston, Massachusetts area. Informed consent was obtained verbally (for interviews) and electronically (for the survey). All study activities were approved by the Mass General Brigham Institutional Review Board.

Community-based methods were utilized to recruit participants, including posting study flyers in online TGD groups, tabling at TGD events, sending email blasts to LGBTQ + organizations and providers with requests to place flyers in waiting rooms, and obtaining peer referrals. Participants were purposively sampled [36] to ensure a wide range of experiences across gender identity and race/ethnicity.

Interviews were designed to be 60 min and conducted via Zoom by a TGD-identified team member (MQ). Interviewer-interviewee concordance was designed to center the shared experiences of TGD people and promote empathy and rapport-building in the interviewing process [37]. Participants were asked to be in a private and safe space where they could speak freely about their experiences. Participants were asked to provide a pseudonym to be used in any publication; if a pseudonym was not selected by the participant, one was selected by the study team (3 participants requested the study team select a pseudonym on their behalf). The interviewer took detailed notes during the interview process to aid in codebook development. Zoom’s automatic transcription and recording features were used and files were stored securely for analysis.

### Data collection instruments

Data were collected using a semi-structured interview guide developed for this study, informed by a review of existing research and input from TGD-identified staff and gender-affirming care stakeholders with expertise in clinical care (see Supplemental Material Table 1). Participants were asked to describe prior healthcare experiences since first realizing they were TGD to the present day. Probing questions were used to understand how experiences might have been improved. Participants also provided feedback on current care models, additional ways healthcare providers and systems could support their needs, and ideal gender-affirming healthcare delivery. Participants completed a brief sociodemographic survey designed for this study including age, race/ethnicity, gender identity, recent gender-affirming care history, education, and insurance status (see Supplementary MaterialTable 2). Survey data were used to descriptively characterize the sample and contextualize interview data.

**Table 1:** Demographics of transgender, nonbinary, and gender diverse participants (*N* = 27)

Demographics	N
**Age, Mean (range)**	28.5 (18–45)
**Gender identity**	
Transgender man	7
Transgender woman	6
Nonbinary	8
Genderqueer	3
Agender	1
Did not specify	2
**Sex assigned at birth**	
Female	18
Male	8
Choose not to answer	1
**Race/ethnicity**	
Asian	2
Black/African American	6
White	13
Hispanic/Latino	2
Multiracial	2
Choose not to answer	2
**Insurance Status**^**a**^	
Medicaid or MassHealth	7
Medicare	4
Private insurance	18
Other, did not specify	1
**Education**	
Completed high school	1
Some college or associates degree	10
Completed 4-year college/bachelor’s degree	10
Some graduate school	1
Completed graduate school	5
**Attempted to receive transition/gender affirmation related care in the past 6 months**	
Yes	18
No	8
Choose not to answer	1

^a^Participants could select more than one insurance type if they had multiple insurers

**Table 2:** Quotes from transgender, nonbinary, and gender diverse participants

**(1) Affirms** **patients in** **individual** **interactions**	Uses chosen name, pronouns, and other gender-affirming language	1. Intake forms tend to be my biggest pet peeve … I tend to judge how in interaction is probably going to go after that based on how intake forms are. -Hayden (they/them) 2. Intake forms that are inclusive [matter]. And actually making use of the intake information that you get, as opposed to just collecting it and then not making use of it at all. -Evan (they/them) 3. Honestly, I feel like the biggest gaps that I have seen throughout different experiences, is oftentimes the intake form process, or the experiences of being checked in for appointments [by] the front desk staff. I feel sometimes they get left out of these types of either trainings or conversations. That’s where I feel like a lot of assumptions are made or misgendering happens. -Hayden (they/them) 4. “Oh, I see you’ve asked me for my preferred name and then stuck it down in a tiny little footnote, while my dead name is big across the page.” Having an actual space that is explicitly there to give your preferred name, then actually using that and respecting it and treating the patient’s dead name as if it were a very confidential piece of information. -Vee (sea/seas)
	Has other TGD people present	5. Being in the waiting room and seeing other transgender people makes a difference because it can kind of get scary… see[ing] other [trans] people in the waiting room makes a difference, too. -Ace (they/them) 6. Creating a culture where other trans people and queer people are there. If I’m scanning the room and I’m the only one who looks or sounds like me with my presentation, that’s gonna be a red flag. -Lyle (he/him) 7. Someone from the community [being present] would be helpful. Maybe not all the persons in surgery because we cannot guarantee that. But someone that represents, it can be like a staff member. -Lucrezia (she/it)
	Eliminates transphobia from and inappropriate verbal and nonverbal interactions with providers	8. It went pretty well until he started talking. [He] went on a rant about Caitlyn Jenner, calling her Bruce Jenner and being like, “Isn’t it weird how like Bruce Jenner is now a woman… he, he, he was such a great athlete…” I said to him, “Can I ask why you are not calling her, you know she/her pronouns, and calling her by her legal name, which is Caitlyn…” He kept going on a rant, “You know, Bruce, Bruce was so fascinating,” and clearly I was pretty uncomfortable, but I don’t think he seemed to care. –Sarah (she/her) 9. They had me meet with one of the medical students before my surgeon came in. I had never met this person before. The very first question that he asked me was if I regretted it. That really put me off. What does this person think about trans people and trans care? What an assumption to make! It felt so shitty because I was finally feeling good about myself, and this person comes in, and is assuming that I regret it… I don’t think it was done with malintent. I think that it was a product of a lack of training, a lack of working with trans people, and knowing all of the hoops that trans people have to jump through to be able to get to that point. -Hayden (they/them) 10. I tried to explain to [my doctor] that I had feelings of powerlessness because I was a woman. This shows you how he didn’t get it. He said, “Well, yeah, you have power. You have power to make men do things for you.” I damn near barfed. -Aaron (he/him) 11. I can tell when a medical provider it thinks that I’m disgusting or thinks of my body as repulsive… I feel like some of them feel we can’t tell and I just want them to know we can tell. -Sarah (she/her)
	Recognizes TGD patients as experts and offers centering goal-oriented gender-affirming care	12. Ideally, you wouldn’t have to wait because how long do you want to be suffering before you don’t? The thing is most trans patients come to a practice know that this is what they want. They figured that out. You don’t accidentally stumble into a consultation. You go there on purpose. By the time that you’ve made that realization it’s not the sort of thing where like the wait serves any purpose. It’s just, you know an extra week of suffering, or an extra month of suffering, or an extra y’ar of suffering. -Vee (sea/seas) 13. People know their own gender, and the only way that other people can gain information about that is by the original person talking to them. The healthcare system seems to have a backwards idea of “they can know your gender.” -Vee (sea/seas) 14. [My doctor] was very much like you get to drive the car here. I’m just here to make sure that your blood levels, cholesterol, and all those things are okay. Otherwise, if you want to up your dose and it’s safe to do so, you can do that. If you want to lower it, you can do that…I was really worried coming in as a non-binary person, if they were going to be kind of like binary assumptions put on me. But I was able to just tell her, I wanna go on a low dose, I don’t want to have this happen really quickly.—Sarah (she/her) 15. I wanted to get a 0 depth vaginoplasty. That was something that I had put thought into, but not after going back and forth on a few different things. The doctor who I was speaking to at the time said, “Well, if you’ve been going back and forth on a few different things, then you should wait.” -Vee (sea/seas) 16. [People say] “there’s been some studies done on menopausal cis women that said taking progesterone increases your risk of cancer by like 15 in a 1,000 or something and so we can’t do that.” I can understand how that how calculation would seem reasonable to someone who is not currently living in my body. But for me the difference is a slightly increased risk of cancer. On the other hand, is living without emotional depth, living without a libido or living with severe genital pain. These are problems that I think are extremely serious when you're living with them to the point where I would do almost anything to have full emotional range -Sarah (she/her) 17. I was researching online. I told a [doctor] from my research online that most people start on this dose of testosterone blocker and start on right away on estrogen. She goes, “No that’s not our protocol.” They didn’t follow any particular protocol- like one from WPATH. She was like, “We see how low [the blocker] gets your testosterone then we introduce estrogen in 4 or 6 months.” That makes no logical sense because [blockers alone are] not doing anything for me… It didn’t make sense to me, especially what I saw online and what I researched on my own. I also joined a bunch of forums to talk to different trans women to see what they are on… I did not go back [to that provider]. -Susan (she/her)
**(2) Flexible and** **accessible**	Makes gender-affirming care available beyond urban population-specific clinics, offers care at non-traditional times and non-traditional settings, and addresses challenges with proximity to care	18. I don’t wanna go downtown [city] for an appointment. I live in [suburb], but I also don’t want to go too far outside the city either. I’m really picky about doctors. I mean just from being burned in the past by doctors, but also too just like you know it’s [city]. I can’t choose who I want to see. I don’t have the luxury of being able to have a choice. -Lyle (he/him) 19. The only downside was just having to travel into the city. I was living in [45 min away]… I had to sneak out of work early. I wasn’t out at work. I would make excuses and come up with reasons I had to leave early. -Link (they/them) 20. There are other surgeons that I’ve seen that have just been too far. I’ve consulted with this surgeon [on the other side of the state]. That’s too far. I wasn’t thinking about the location when I made the consult. I also booked a consult was someone in Florida. Obviously, location is too far. Then, also with someone and UK which is obviously way too far. -Dan (he/him)
	Reduces wait times through facilitated linkages to gender-affirming providers	21. I did talk to somebody about a vaginoplasty to see how long it was, and it’s like a 3 year wait… I’ll fly somewhere to do it before I wait 3 years! -Susan (she/her) 22. When I realized that I needed a new pcp [primary care physician], I was like “Oh, I can’t just go and find one who’s accepting patients. I want to find one who I can trust, and who can speak to my health concerns, not just [my] general health.” …I’m learning more about where to start looking but especially in the [city] there’s just so many doctors. I feel like most of them would say “I’m LGBTQ friendly” because it would be hard to be a doctor in [city] and be like “I am homophobic.” But that feels different than “I am a doctor who is also capable of providing specific care and health information for someone who is trans and gender diverse.” … I don’t know how to narrow it down…I don’t want to do the work of going and meeting them and then finding out that they don’t make me feel good. -Sylvi (they/them) 23. I was receiving hormone replacement therapy. Eventually I just got sick of dealing with [local LGBTQ health clinic], because they kept referring me to different providers, because providers would leave. I just really don’t wanna deal with that all the time. -Tom (he/him) 24. Unfortunately, [the local LGBTQ health clinic] is really swamped all the time. It’s almost impossible to get an appointment. I need an eye exam and it’s a 9 month appointment wait. I’ve been trying to get therapist and psychiatry there. I’ve got a lot of mental health issues and learning, disability and some other stuff that needs constant psychiatric and therapeutic care. It’s been very hard to get that. I eventually got a therapist, but then she went on vacation for 4 months straight immediately after my intake appointment. I’ve been waiting about a year for that, and I still haven’t seen a psychiatrist yet. -Sarah (she/her)
	Improves disability-related accessibility for TGD patients with disabilities	25. There is an overlap between people who are queer and people who are disabled or neurodivergent in some way. So having a room that is autism and ADHD friendly. You know, having doors that are accessible, and a bathroom that is accessible it makes sense. Having lights that aren’t those terrible fluorescent lights that buzz really loudly. Sometimes, oftentimes the queers be neurodivergent. -Armani (they/them) 26. You would think medical providers would be understanding about certain things, but they’re not always accessible spaces. Sometimes I use a wheelchair to get around, and I want to be able to take my wheelchair to the doctor. -Lyle (he/him) 27. There’s so many like requirements that I have to watch out for with the bleeding disorder in regards to top surgery… I need to make sure that the hospital that they have permissions at is capable of treating the bleeding disorder. I also need to make sure that [the surgeon is] not too far in case I have a hematoma. -Dan (he/him)
	Increases financial accessibility of gender-affirming services	28. My partner at the time came from a very well-off family. She ended up paying for the majority of [top surgery]… I started paying her back as I was able to. I was in a very fortunate place to have that because otherwise I wouldn’t have been able to. I definitely would have had to go the insurance route or wait a lot longer to be able to save up… she ultimately broke up with me because I’m trans… initially, I felt like- how am I ever goingto love my body knowing that she played such a role in getting me to that, but then ultimately didn’t want to be with me because of that. Just a lot of mental gymnastics…—Hayden (they/them) 29. I recently had a consultation for FFS [facial feminization surgery]… The cost is astronomical so I’m work with my employer to see if we can get any policies on the books for trans health specifically… I’m gonna have to do a lot of research, talk to a lot of people, figure out how to go [about this]. I’m steadily learning that you really have to be your own advocate, and you really have to become an expert to navigate the world of healthcare as a trans person. -Link (they/them) 30. Medication technically doesn’t expire. It loses a little bit of potency. The needles don’t expire… if I’m ever out of work and I can’t afford the copay I have it on hand…I go through [pharmacy] for some stuff because it’s a lot cheaper like finasteride is not covered by insurance. If I go to another pharmacy, it’s like a $150 for 90 pills [of spiralactone], but if I go to my pharmacy it’s 12 bucks… You gotta shop around for your medications, but it’s just a pain to have so much on hand. Yes, I’ll go through the spirolactone. But the estrogen injections, I take it, every 2 weeks. It takes me 2 months before I get through a full vial, so it’s kind of a waste of estrogen – I hate to see it expire…I’d rather have it than not have it and then be out or have a crises. -Susan (she/her)
**(3) Fights systemic** **oppression**	Dismantles gatekeeping practices related to gender-affirming care	31. I think probably the top thing is just like how incredibly hard it is. Because I feel like until you’ve gone through the amount of fighting it takes it’s really hard for people to understand what it’s like to have to fight for access to being who you are in your basic identity. And so when they when people say like oh, it’s no big deal for you to get like a letter from a provider saying like you can do these things, or like verifying you are who you are, it just feels like it’s that’s a person who has no idea how incredibly crushing it is to go see a provider, and them like say no to something or them like, say something that makes you feel not okay. And I think that like a lot of the a lot of our medical care, just sort of writes off the amount of like time and energy and fight all of that takes in order to do any of that. And then expects us also to like, be really good at dealing with insurance, and take really good care of ourselves, and navigate, being the one trans person out of our workplace really well, and not be depressed. -Hellen Highwater (they/them) 32. After maybe 3 or 4 months seeing [a therapist], she recommended that I go to an endocrinologist so I could start gender-affirming hormone therapy… That was my first experiencing gatekeeping. I had to wait 3 months to get an appointment… I was very nervous. The doctor was nice and cordial but very blunt…But afterwards when I played it back, I realized he was treating me in such a way where he wanted to get a sense of me in 2 min and feel if I met his criteria to give me hormones. It was a very dehumanizing experience. He ended up saying no – even though I had a referral from my doctor and I was diagnosed with the gender dysphoria through my therapist. He told me that… I had to come back in 3 months… I cried the whole way home. I really beat myself up because I thought maybe if I was able to explain myself better the experience would have gone better. I came back 3 months later. I realized I couldn’t show that guy any doubt. I had to give him a story that he could understand. So I pretty much told them what he wanted to hear. Got my hormones, got out. -Link (they/them) 33. If you aren’t going through insurance and providers are still requiring a letter that’s still sending this message that they like need to protect themselves for reasons that are related to people thinking that people are gonna regret it. Thinking that people are going to detransition, or high amounts of people, are going to detransition. Whereas like I’ve had knee surgery twice, and I didn’t have to go to a therapist to say, yes, you can have this knee surgery, and so I think it should be the same way for any type of gender-affirming care. Why, are we like gatekeeping these policies when we know that therapy is expensive, that not everyone’s able to access that that there are providers that do act as like strict gatekeepers, and like, why should we be putting people in a position where they like potentially have to be like invalidated in their identities by somebody with more power than them? -Hayden (they/them) 34. When I was really worried about like asking my therapist to write the letter for me. It would have been meaningful had she said something calling out the fact that she is in a gatekeeping position, or recognizing that we’re in a system that values these gatekeeping practices, but that she trusts my own knowing of who I am. I guess just being more open with empowering patients to know themselves and to be able to be experts in their own experiences… I think that agency is really important for providers to explicitly have a conversation about. -Hayden (they/them) 35. I think [I would] mostly [want] access to a nonstandard surgical procedures. And when I say nonstandard, I really mean anything other than metoidioplasty, phalloplasty, vaginoplasty. As far as bottom surgery procedures go. In doing research for that back many years ago, when I was still trying to figure out what I wanted, the number of places where you can even just find information about those procedures is really limited. You’re looking through each hospital would have maybe one page on it, and there’s only 2 hospitals who have pages on it, and you’re just flipping back and forth for these in the surgery that you want to have, like maybe a paragraph about it. Which just is unfortunate. I feel like having explicit access to those services. -Vee (sea/seas) 36. Trans Bucket is a website where people post pictures of their surgeries results… The New York Times came out with an article like shitting on phalloplasty. They literally called it a Franken-weenie, which is absolutely disgusting for them to do. They mentioned Trans Bucket, and then a bunch of conservatives hijacked it… It became an unsafe website. Now it’s shut down, now you can’t really find those surgery results that you need [to see]. -Dan (he/him)
	Understands intersectional experiences of TGD patients (e.g., racism, cisgenderism)	37. I didn’t feel safe in [other state] because of my gender and before that because of the color my skin, like I needed more diversity community and medical support. -Ace (they/them) 38. I’ve talked about this with my friend who’s black but cis. Medical providers often don’t listen to black people. [Providers] don’t believe them and sometimes I feel like there’s an internalized pressure for myself… related to socialization as an Asian person, or this expectation of Asians not really speaking up you know. It’s not necessarily that this is what providers have told me but it’s more of a societal thing… it’s tough because I think it just in general the whole medical provider complex thing is just weird because they spend 5 min with you and they’re like, “Okay, Are you done are you done? Are you done? Do you have any more questions? No? Okay, any more questions? Bye!” They just wanna get out of there.… but it also doesn’t help that [city] just feels really racist in general… So do I really want a provider who’s also super white? -Doc (no pronouns) 39. I told [the same PA] specifically, “Hey, there was a person like at the reception desk who is like super white and super loud. I need you all to like tone that down.” [The PA] was also black. We could relate on dealing with racism as trans people. They actually went and figured it out. The issue was that they were standing far away from the desk, because of like the 6 feet thing. That person was yelling and I was standing over here trying to talk to my person. I’m having to repeat myself through a 1 million times and being Asian and having the stereotype of being we’re quiet… it made me feel really uncomfortable… it was all these kind of oppressions relating. -Doc (no pronouns) 40. It’s complicated because I at least want someone who is BIPOC who looks like me honestly identifies as me I feel like that would help so much. When I was in high school, I had a counselor, and she was exactly like me. She was Hispanic, female, and lesbian, so she’s pretty badass. I loved her… But it’s hard because they provide at least like who the providers were- you can see pictures and stuff. But all the BIPOC and LGBTQ ones are taken up for such a long time, and they’re so little of them. It’s like a little lottery. -Buttercup (any pronouns)
	Navigates complex power dynamics and medical culture to ensure TGD patient-centeredness	41. I hadn’t really shared anything about being trans with my previous physical therapist,because didn’t feel like a safe environment. It was very bro-y. -Hellen Highwater (they/them) 42. I didn’t want to say anything in the moment because doctors are scary and have a lot of power. But at the time I didn’t think about [my care] as something that could be different. I just assumed that this is what I’m gonna have to deal with… It wasn’t until later conversation with a friend when I realized that doctors could ask about sexual orientation and gender identity. I was like, “Oh, why don’t why don’t they all do that? That would be so helpful!” -Sylvi (they/them) 43. I was in a hit and run with a cop where the cop knocked me off my [motorcycle] intentionally… I was bleeding all over the place. I had to wait for the cops when the ambulance brought me in the emergency room and right away… When they finally realized I’m not dying, they brought me into another room where now they have male doctors come in and tell me to take off my shirt and clothes. I said I’m not taking off my shirt in front of you. [They asked,] “Well, why?” I go, “That’s none of your business why.” It was almost a forced thing. They had a woman come in to help me. Then she sent him back in while I was still getting undressed… It was traumatizing because just what happened, and that was traumatizing in the hospital how I was violated. Then after they needed my room. They took me in a Johnny with nothing else on and then put me in the hallway with other patients. It’s like how about no? I requested that they give me my clothes cause I’m leaving. Bleeding or not I want out. You know I’ve left the hospital a few times before the way they’ve treated me. -Hellen Highwater (they/them)
**(4) Interacts with community**	Creates TGD Community Advisory Boards	44. My first answer [about a CAB] would be yes, of course, that sounds wonderful! My second response would be I don’t really understand what the downsides of that would be… it would give [you] a way to get feedback on a lot more of the institutional level stuff as far as developing a mission and refining it …. It would offer a group of people that are committed to trans healthcare without putting the burden of that work on all trans people… People can self-select that might be particularly interested or impacted by the work that you’re doing. -Sylvi (they/them) 45. It sounds like a great idea in terms of getting a sense of the people within the community, their views and experiences, and what they want. I imagine it’s similar to the idea of a needs assessment. You can provide all you want for what you think a community needs but until you know what the community actually needs you’re not necessarily going to be providing the best care. I imagine through these CABs it would give a better sense of hearing the voices of the community and knowing what they’re hoping for in order to provide back to the community in the best way. -Evan (they/them) 46. I mean they’re the folks who are the consumers of the health care. They’re the ones that know … they know their condition better than any doctor would so they can advocate for what they need. It’s also good to have that collaboration -Tom (he/him) 47. I think it could be helpful, certainly couldn’t hurt. I wonder if measures like that do as much as they’re expected to. It seems to me that the people who really need to be informed by it are the ones who aren’t even listening in the first place.’ -Aaron (he/him)
	Shows up to be present in and Sponsors TGD community events	48. I found this study at the trans resistance march. The presence of medical healthcare facilities there was really cool. I was like, “Look at all these people that are like in some way or another committed to trans healthcare!” That’s cool. I saw [a local LGBTQ + community health center] there. It prompted a conversation with my friends about how [they are] a great resource. Now I have a name of another place. -Sylvi (they/them) 49. I keep seeing various healthcare providers having booths at pride events and such. That’s definitely a way to get visibility out. -Aaron (he/him) 50. What about a clothing swap? I know [local LGBTQ + community health center] does that. I’ve gotten some good stuff there. I’m not sure if this would actually raise the profile of your program or just get a bunch of people a bunch of cool clothes. But either way, any publicity is good publicity. -Aaron (he/him) 51. [Community health center] did a little support group, which was a really interesting way to connect [with other] patients and to weave medical care into a community-based thing…—Jack (he/him) 52. I have gotten a lot of targeted ads on Instagram for trans healthcare… also, [talk to] all of the universities in [city]. The school I was at had some network that they could connect people with. Having a stronger relationship with the on-campus health centers would be an effective way [to connect with community] cause a lot of students stay in the area. Once I graduated, I couldn’t get a letter of support from psychiatrist that my school, but now I know how to connect to the [other places] because I saw [other places] on [my school’s] website or met them at a health fair. -Sylvi (they/them) 53. Maybe [try] a collaboration with a library to talk about the history of trans health, looking at oral histories of trans people in [city], or with the history project about how queer community has grown in [city] and how health is a part of that. Less about health care provision, and more about the history of healthcare kind of things. -Sylvi (they/them) 54. They can make events for transgender people to feel more welcome… For example, if there’s an event where it is for LGBTQ kids- have books to read them about LGBTQ + people. I really feel that if they have these short story times, they will feel that kind of representation, and also they will feel safer in the hospital. -Lucrezia (she/it)
	Addresses holistic gender-affirming care needs of TGD communities (ensures positive “word of mouth” communication in TGD communities)	55. A lot of the time when it comes to healthcare within the trans community it really comes by word of mouth. Even if something is advertised, without knowing if someone’s experience is positive or negative, people are going to be hesitant to go. Part of it is trusting time, in terms of like, if someone is already going or utilizing these services and having a positive experience, they’re gonna tell other people about it. You can do all the advertising you want, that will get people’s attention. But at the end of the day, prioritizing people’s individual experiences and trusting that they’re gonna tell other people is one way to get the word out there 56. To an extent, you need to get your name into the circles, but no amount of advertising, handing out flyers, or letting people know will really matter if trans people have bad experience with it because they’re not gonna recommend it. If they have a better experience with some other place, they’re gonna be like, “well, I’ve gone to these two places, but I wouldn’t recommend you go to this one. I would go to this one.” -Vee (sea/seas)
**(5) Retains patients** **in care**	Recognizes the need for trauma-informed gender-affirming care	57. [Social media creator] is very transphobic… I was put on blast by him… Somebody spread my information and I was attacked by him. I had tens of thousands of emails messages. My Facebook has been bombed. I had people [standing] outside my [place of] work. I’ve been protested at my job. I’ve had people harass me before I go into my job. It’s an ongoing thing. I’ve had to endure a lot… we’ll leave it at that. It’s been to the point where you know I had thos’ dark thoughts again… [I’ve] called the suicide hotline twice, because of this. [There’s] only so much you can handle, you know. -Susan (she/her) 58. It does have to do with abuse. They thought they could get away with it and that I deserved it. But that’s all I’m comfortable sharing. -Zena (they/them) 59. I ended up being sexually assaulted by [my doctor] during the consult. -Dan (he/him)
	Builds and rebuilds trust with TGD communities	60. I needed care that was unrelated to being trans. I had a concussion, so I just needed to see someone. To get in you have to wait 3 months. That’s just ridiculous. That’s not specific to being trans but it just kind of adds to the stress of being trans in general. Like hey, I have this health thing, and I’m already not super trusting of any kind of providers… it just makes things tougher. -Doc (no pronouns) 61. I mentioned [challenges with my mental health] to my health provider maybe 2 or so years ago. I really wanted to learn more about mental health providers… I was open with them because… they’ve known me for so long. I built that trust with them. That’s why I was able to mention it to them. They were very understanding and very kind of about it. They understood and they gave me those resources. -Buttercup (any pronouns) 62. I graduated from my pediatrician a few years ago and have put off finding a new doctor because it seems like a lot of work to find a doctor that I actually trust. -Sylvi (they/them) 63. I have a lot of trust in doctors to do the right thing for health. But then the comfort part always felt totally secondary… it felt like they were assuming that I would be comfortable with everything because they saw me as a man. -Sylvi (they/them) 64. It’s not just about the tissues there, the tissue’s gone, and that just didn’t feel an actual genuine understanding of what he’s trying to do as a surgeon and the relationship to the trans community. I was not, even though he could have gotten me in sooner, comfortable proceeding with him. He just had that attitude, and honestly [he] felt like a used car salesman and refused to show any results at all. -Hellen Highwater (they/them) 65. A doctor will use some language that I really would rather not be used to refer to me, but honestly, that would be that’s a pretty small thing for me. It does me off of the doctor and the hospital in it, certainly makes me trust them less, but it’s not verbal abuse or anything—Vee (sea/seas)
	Provides TGD patients with gender-affirming care referrals for unmet needs	66. It means that you understand that just because I feel safe telling you about my health problems doesn’t mean I’m gonna feel safe with the specialists that you refer me to. Lyle (he/him) 67. One weakness that [health center] has is that they can refer me to a urologist. But they don’t know which ones are trans competent. So I have to do a little leg work. -Aaron (he/him) 68. One of the things that I didn’t like about [LGBTQ + health center] is that I was constantly being referred out for care. My needs are kind of complex, but I’m also human. There’s gotta be other people that also have these problems. Diabetes is not really uncommon and you don’t really know how to help me? -Tom (he/him) 69. You have to see the first doctor. Later you have to see another doctor. I just want to make sure like how quickly the referrals are. Because I really feel that if you know exactly what you’re going to do, why do you have to see so many people to go to the one that you want? I really want to make sure that the referrals are done quickly. -Lucrezia (she/it)
**(6) Multidisciplinary**	Builds integrated and connected health care teams, incorporates non-medical professionals (e.g., peer navigators), and addresses broader TGD health-related needs (e.g., social services)	70. I mean imagine you’ve got patient advocates or ombuds people… I wonder if I wonder if part of [health] network[s] could be one of them. Someone who knows how to advocate for trans people -Aaron (he/him) 71. I think supports around the name change process. I know there’s information online, but some people need someone to go with them to advocate or to sit with them because it can be intimidating. -Tom (he/him) 72. [My PCP] knew absolutely nothing about top surgery other than it existed. She was willing to write a letter after like she requires me to have a conversation with her like a person-to-person conversation. I couldn’t just write or an email and say I want a referral for top surgery, even though she knew that I identify as non-binary. But I’m cool with that that seems like due diligence to at least have a meeting, and it was just over zoom… But she was not at all prepared to actually write the letter. She didn’t have any idea what to put in a letter. I ended up having a social worker friend of mine write the letter and sent the full text to my PCP saying, “Can you review this? Change anything you want to change. Put it on your own letter head and sign?” … She was very happy to sign it. -Hellen Highwater (they/them) 73. A lot of it- changing your identity, license, social security -and the thing is support services that I never heard of. For example, I’m not sure if you ever heard of a [local mutual aid fund]… They’ve been a very great community resources like that. Nobody tells you about, you know they provided me a month. I was really late at my house, they provided me a mortgage payment for a month, you know. There’s like resources like that housing isn’t seems to be another big issue with transgender folk is a lot of people don’t want them in their housing. … You know, where are these resources other than sit there? If you don’t have access to the Internet, like I do, where do you find them? You know there are places out there that will give you help paying for your name change. There are places out there that give micro grants for changing your social security. This stuff is not cheap. They will help, you know, some place where, even if you were to not have it at a site, you could have it on internal like website where you have a list of links and resources. And then you can say, Okay, go here or you could print them up. Okay, here’s a list of places you can contact and just give phone. Just have something other than the traditional okay well here’s your STDs. You can get AIDS, you can get syphilis, you know. I I know all that. -Susan (she/her)

### Data analysis

Upon interview completion, Zoom’s automatic transcripts were compared to audio files and edited by SB and AM to be verbatim. Any potentially identifying information was redacted, Interviews were double-coded using an immersion-crystallization [38] and a reflexive thematic analysis [39, 40] approach. Dedoose software [41] was utilized for analysis. Throughout the interview, transcription, and review process, the team immersed themselves in each of the interviews. MQ, SB, and AM developed, iteratively refined, and applied an initial codebook that emerged through the immersion process to all interviews. The analysis team met weekly to obtain consensus between coders, iteratively refine themes and subthemes, and collaboratively consolidate and organize codes with a focus on reflective and reflexive engagement [39, 40]. Meetings were also held with members of the investigative team to discuss and refine codes. Representative quotes were selected for reporting. For readability purposes, filler and repeated words were removed from quotations.

### Positionality statement

Interviews were conducted by a TGD team-member, which allowed for rapport building with participants. The analysis and reporting process were completed by a primarily TGD-identified team with support from cisgender researchers and gender-affirming clinical providers. The team was comprised of Asian American, Latine, Indian, and White American individuals. Multiple team members have navigated medical systems both as TGD-identifying people and chronically ill individuals. The analysis and interpretations of this study are informed by our positionalities, identities, and experiences.

## Results

### AFFIRM Framework overview

**Fig. 1:**
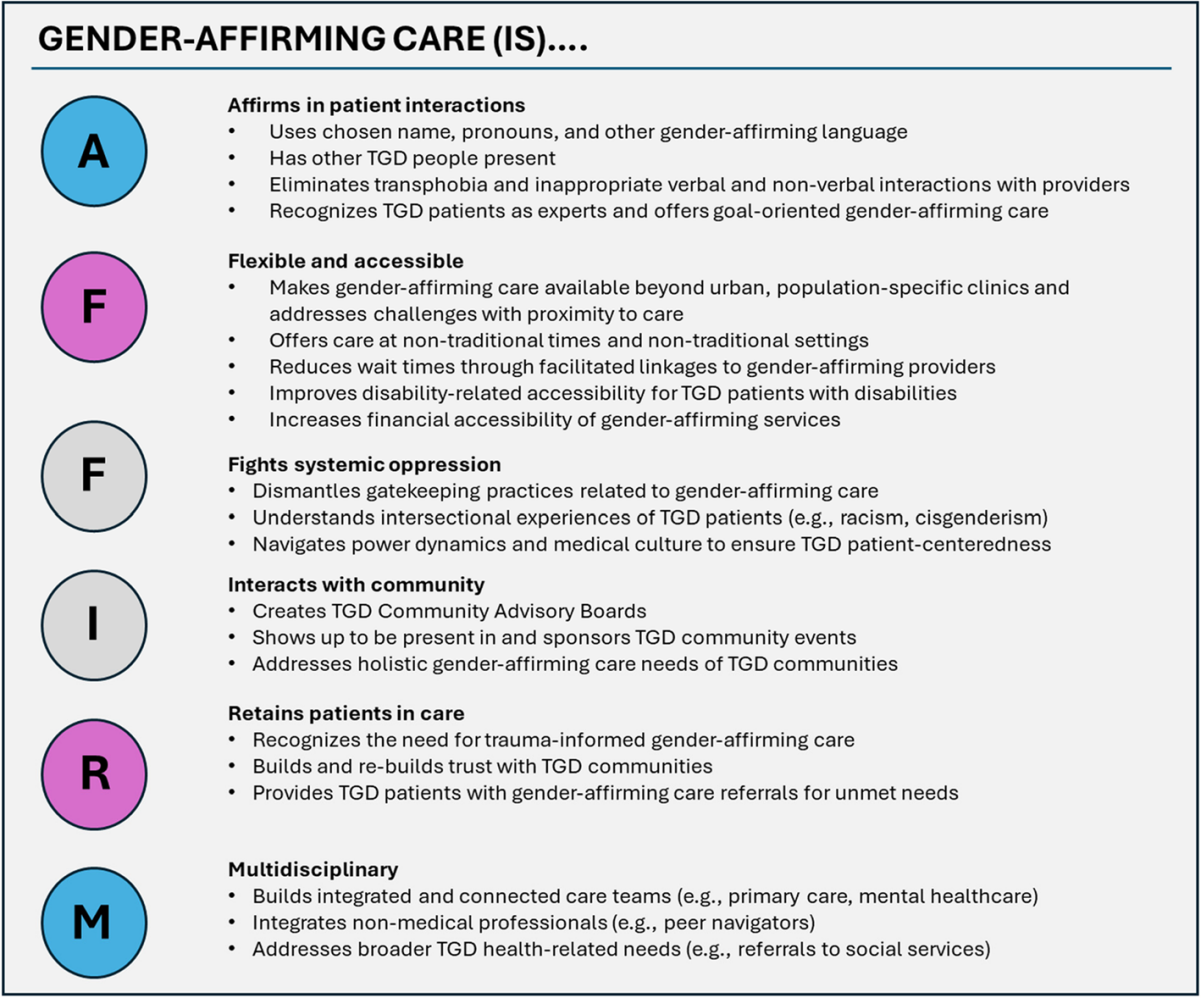
Overview of the AFFIRM Framework: Affirms in individual interactions, is Flexible and accessible, Fights systemic oppression, Interacts with community, Retains patients in care, and is Multidisciplinary. TGD = transgender, nonbinary, gender diverse

Participants (demographics in Table 1) shared stories of both affirming and disaffirming healthcare experiences. These experiences occurred in seeking gender-affirming medical care (i.e., medical care explicitly related to transition/gender-affirmation, such as hormones or surgery) and other medical care not explicitly related to gender-affirmation (i.e., healthcare that is gender-affirming). Needs coalesced into six main actionable components for gender-affirming care as described by the acronym AFFIRM (Fig. 1): (1) Affirms in individual interactions: Participants called for affirmation of TGD identity, lived expertise, and experience from TGD providers and staff. (2) Flexible and accessible: Participants expressed the need for gender-affirming care to be available beyond urban, population-specific clinics and in a timely (e.g., no year-long wait list) and community-centered manner (e.g., non-traditional healthcare times and settings). (3) Fights systemic oppression: Participants emphasized the need for providers and health systems to eliminate gatekeeping practices for gender-affirming care and create models of care that resist oppressive systems, including intersectional oppression (e.g., racism, cisgenderism). (4) Interacts with community: Patients desired intentional interaction with TGD communities to holistically address health and unmet gender affirmation needs. (5) Retains patients in care: Patients shared the need to collaboratively identify and problem-solve obstacles to gender-affirming care with providers and healthcare systems to optimize TGD-specific retention strategies. (6) Multidisciplinary: Patients called for teams that are interdisciplinary (e.g., integrate primary care, mental healthcare, letter-writing for surgical care) and that incorporate non-healthcare professionals (e.g., peer navigators) to meet the broader health and wellbeing needs of TGD people (e.g., social service needs).

Table 2 contains sub-themes that comprise AFFIRM and all participant quotes. Participant quotes are identified by numbers in brackets throughout the Results. Pseudonyms are used throughout the manuscript for readability.

### (1) Affirms in individual interactions

#### Uses chosen name, pronouns, and other gender-affirming language

Participants discussed affirming language as a priority and highlighted the importance of asking names and pronouns on paperwork and using these in interactions with front desk staff “to judge how the interaction will probably go after that” (1). Some participants shared that while this information was initially collected, providers and their teams often failed to “actually make use” (2) of the information. One participant, Hayden, noted that providers and staff must be trained to use this information (3). Vee suggested that deadnames, or names people no longer use, be treated as “confidential information” (4). Participants noted that having their legal names easily accessible in electronic health records (EHR) for insurance and billing purposes resulted in it being misused by providers and support staff.

#### Has other TGD people present and active allies

Participants described being the only TGD person in waiting rooms can “get scary,” and that having other TGD people around increased their comfort (5). Lyle said that not seeing other queer people was a “red flag” for them (6), potentially indicating that an office might not be affirming of TGD people. Participants were interested in having a TGD-identified provider, but Lucrezia recognized that there are few in medicine (7). Lucrezia also stressed the importance of having TGD staff present in care settings.

#### Eliminates transphobia and inappropriate verbal and non-verbal interactions with providers

Most participants reported hearing transphobic sentiments from their providers including intentional misgendering of public figures (8), inappropriate inquiries regarding surgical regret (9), and sexually charged statements (10). Some participants felt these were intentional, while others did not. Hayden felt that statements by their provider came from a place of ignorance, but the painful moment “stuck with them” regardless of intent (9). Body language and other nonverbal cues were also discussed. Sarah stated, “I can tell when a medical provider thinks that I’m disgusting” (11). Rather than being covert, body language and other non-verbal cues from providers were described as overtly communicating transphobia.

#### Recognizes TGD patients as experts and centers goal-oriented gender-affirming care

Many participants expressed wanting providers to trust that they had “thought about things” and were experts in their own gender experience. As Vee noted, “You don’t accidentally stumble into a consultation” (12). Vee also remarked that you can’t “scan [someone’s] brain and know their gender….and the only way that other people can gain information about that is by [talking to] the original person” (13). Sarah appreciated that their provider allowed them to be the “driver” in their affirmation journey, and felt the provider just helped them to drive the car “safely” (14). In contrast, Vee described a provider who perceived the patient as “going back and forth” about seeking gender-affirming care, which resulted in denial of care (15).

Many participants wanted providers to ask them about their gender-affirming care goals. Several participants spent time researching risks and benefits of gender-affirming care and determined that their transition needs greatly outweighed potential risks (16). Nick expressed challenges with the speed and manner at which his provider wanted to go about transition, which did not align with update-to-date medical guidelines or with his peers (17). Treating patients as experts in their own experience and asking about goals were highlighted as essential for gender-affirming care.

### (2) Flexible and accessible

#### Makes gender-affirming care available beyond urban population-specific clinics, offers care at non-traditional times and settings, and addresses challenges with proximity to care

Finding affirming providers who were in geographic proximity and having clinical care outside of traditional work hours were described as unmet needs by multiple participants. Participants seeking non-surgical care often had to travel into Boston for care. Lyle described needing to travel far distances to get care saying, “I don’t have the luxury of being able to have a choice” (18). Link made up excuses to sneak out of work early to be able to travel for care (19). Participants seeking surgical care had even greater challenges finding competent surgeons nearby resulting in interstate and international travel (20). Many participants expressed frustration about trying to find affirming providers within reasonable geographic proximity.

#### Reduces wait times through facilitated linkages to gender-affirming providers

Wait times and finding affirming providers with availability resulted in significantly delays in care. One participant was on a three-year waitlist at a local provider for vaginoplasty and ultimately sought care out of state (21). Sylvi described difficulty figuring out who would be affirming, given many providers who list themselves as “LGBTQ friendly” may not have the actual skills to support TGD care (22). Other participants discussed affirming care experiences, particularly in LGBTQ + clinical settings, but noted high provider turnover as a challenge in maintaining care continuity (23, 24).

#### Improves disability-related accessibility for TGD patients with disabilities

Some participants discussed the overlap between queer identity and disability, noting the requirement that healthcare spaces be accessible for physical, developmental, and sensory-related disabilities (25). Lyle expressed challenges accessing healthcare when using a wheelchair (26). Jeff described challenges finding gender-affirming surgeons capable of managing complex medical conditions during surgery, like a bleeding disorder (27).

#### Increases financial accessibility of gender-affirming services

Participants expressed challenges in affording and getting gender-affirming care covered by insurance. Several participants relied on financial support from family or partners. For example, one participant discussed challenges paying back an ex-partner for top surgery (28). Link, a participant with access to insurance, had to become their “own advocate, and… an expert to navigate the world of healthcare” to get facial feminization surgery (29). Participants recognized that this healthcare access advocacy would benefit them and others but noted that it required time and was draining. One participant stocked up on medications in case the copay was raised, or they lost their job, and shopped for cheapest price medications (30), something other patients might not have the time or knowledge to do.

### (3) Fights systemic oppression

#### Dismantles gatekeeping practices related to gender-affirming care

Many participants described gatekeeping experiences wherein providers and systems prevented or made it difficult to access treatment. Participants stressed how “incredibly hard” it was to “fight for access to being who you are in your basic identity” amidst gatekeeping systems inhibiting access (31). One of Link’s providers denied their hormone therapy despite persistent gender dysphoria because of his own assumptions and biases about what a TGD person should look like (32). Another participant discussed the stark differences between pursuing knee surgery versus gender-affirming care (33), highlighting high costs of therapy needed to obtain letters supporting gender-affirming care. The same participant appreciated that their therapist and endocrinologist directly named their “gatekeeping position” and enhanced their “agency” in decision-making (34). Gatekeeping was also discussed in relation to information-seeking. Vee could not find any accessible lay-language information to make decisions about surgeries, other than metoidioplasty, phalloplasty, and vaginoplasty surgeries (35). Another participant expressed frustration because some community websites, where surgical results were shared within community, had been shut down, limiting the available information about surgeon’s results (36).

#### Understands intersectional experiences of TGD patients (e.g., racism, cisgenderism)

Many participants had intersecting marginalized identities and described multiple forms of oppression in relation to healthcare experiences, such as racism, ableism (discrimination and social prejudice against people with disabilities), and transphobia. These participants expressed frustrations in seeking care as a person of color or disabled person on top of seeking care as a TGD person. Ace shared that they moved states because they needed access to a more diverse community and medical support (37). Black, Indigenous, and other People of Color (BIPOC) participants commonly discussed provider stereotypes about patients of their racialized identity group, including racist stereotypes in relation to patients with Black and Asian identities (38, 39). For several participants, having a BIPOC provider for gender-affirming care was a priority. Buttercup summarized: “I at least want someone who is BIPOC who looks like me, honestly identifies as me, I feel like that would help so much” (40).

#### Navigates complex power dynamics and medical culture to ensure TGD patient-centeredness

Multiple participants expressed concerns about power dynamics in medical spaces. For Hellen and Sylvie, this meant staying closeted in a “bro-y” environment (an environment dominated by cisgender men where non-cisgender men may feel like they do not have space) (41), and fearing being open with doctors because “they have a lot of power” (42). One participant was taken to an emergency room after, what they described as, an intentional hit and run by a cop, and relayed the traumatizing nature of the incident and re-traumatization at the hospital when male doctors asked her to take off her clothing in an uncompassionate and uncomfortable manner. She alluded to her discomfort and power dynamics, which ultimately caused her to leave despite still needing medical attention (43).

### (4) Interacts with community

#### Creates TGD community advisory boards

All participants saw potential benefits associated with Community Advisory Boards (CAB) for TGD health programs to share feedback with health providers to iteratively improve health systems (44–46). Despite ultimately supporting the idea of a TGD CAB, one participant was concerned about the marginal difference between what a CAB offers and what is currently implemented at already gender-affirming health centers. Aaron stressed, “The people who really need to be informed by [a CAB] are the ones who aren’t even listening in the first place” (47).

#### Shows up to be present for and sponsors TGD community events

Multiple participants expressed their desire to see healthcare teams attend and sponsor community events. Sylvie heard about the current study through a local TGD rights march, where they had also found a new healthcare provider (48). Aaron saw TGD health programs at other pride events, stating that it was an effective way to “get the word out” (49). Participants suggested hosting clothing swaps (50), holding peer support groups (51), putting up targeted ads on social media (52), connecting with local college health centers (52), sponsoring TGD health history discussions at local libraries (53), and having story hours (54). Lucrezia stated that these events would make people “feel like more safe in the hospital” (54).

#### Addresses holistic gender-affirming care needs to ensure positive “word of mouth” communication in TGD communities

Participants described “word of mouth” in TGD communities. As Evan noted, even with advertising people might be “hesitant to go” to a clinic if they don’t know whether other TGD people have had a positive or negative experience (55). They also stressed the need to prioritize individual patient experiences to keep them coming back. This sentiment was held by multiple participants, including Vee, who wanted healthcare organizations to attend events to get their name into the community, but also highlighted the need to maintain TGD community trust through ongoing, positive individual healthcare interactions (56).

### (5) Retains patients in care

#### Recognizes the need for trauma-informed gender-affirming care

Participants discussed past traumatic experiences outside and inside of medical settings which they carried with them, emphasizing the need for trauma-informed care. One participant described a doxing experience where they were “put on blast” by an anti-transgender online account that “spread [their personal] information”, resulting in receiving thousands of threatening messages, and consequently having a steep and rapid decline in her mental health and considering suicide (57). This participant expressed how they carry this and other traumatic experiences into the healthcare setting, meaning providers must navigate patient’s traumas even when they are not directly related to, but can indirectly impact, their physical health. Evan could not even bring themselves to talk about abuse they experienced at the hands of past providers (58). Another participant described being sexually assaulted by a provider (59).

#### Builds and re-builds trust with TGD communities

Participants described distrust or difficulty establishing trust with providers (60). As a result, Buttercup did not want to transition to adult care and stayed with their supportive pediatrician (61). Sylvi said they “graduated” from pediatric care but “put off finding a new doctor because it seems [like] a lot of work to find a doctor that [they] actually trust” (62). For some participants, TGD-experienced providers experienced garnered their trust, while for others, that professional expertise did not extend to “the comfort part” (63). One participant stated a surgeon’s problematic attitude was a primary reason for not selecting him: “[it] didn’t feel like an actual genuine understanding of what he’s trying to do as a surgeon and the relationship to the trans community… [he] felt like a used car salesman” (64). Vee noted that hearing inaccurate and uncomfortable language eroded trust in providers but discounted it saying, “it wasn’t verbal abuse” (65). Lack of trust resulted in healthcare avoidance or delay for many participants.

#### Provides TGD patients with gender-affirming care referrals for unmet needs

Participants generally indicated difficulties with referrals, including referrals to gender-affirming providers and the structure of referrals themselves. Participants explained how a provider being affirming does not always translate to safe referrals (66), as providers don’t necessarily know which other providers are “trans competent” (67). Some participants discussed issues with referral logistics, such as disliking being referred externally (68), or frustration with the long process and time it can take to be referred to another provider (69).

### (6) Multidisciplinary

#### Builds integrated and connected health care teams (e.g., primary care, mental healthcare), incorporates non-medical professionals (e.g., peer navigators), and addresses broader TGD-related health needs (e.g., social services)

Participants desired care across multiple areas of their health and well-being, including physical health, mental health, and socio-material needs. Participants described the need for “networks” of integrated health services at large hospitals. Aaron desired trans competent patient advocates to support them across healthcare spaces (70). Peer navigation and social work services were highlighted as unmet needs. Participants also saw healthcare teams as a natural place to integrate support for well-being needs beyond physical health, such as “intimidating” name change processes (71). Hellen expressed how their primary care provider did not have the necessary knowledge to write a surgical support letter, so they asked a social worker friend for peer support, who helped guide the physician in letter-writing (72). One participant shared about local support services for housing, name change, and other financial support that few TGD people know about (73), seeing healthcare spaces as prime locations to share resources and provide social support.

## Discussion

This study of TGD adult patients identified multiple unmet needs in the provision of gender-affirming care, extending prior research on this topic to the U.S. context [22, 42, 43]. Our findings also expand upon the notion of community-driven gender-affirming care described in Canada, which emphasizes the need for intentional integration of community stakeholders to shape care and service provision [31]. Consistent with existing clinical guidelines [14], gender-affirming care was not only described as care specific to medical gender affirmation, such as hormones or surgery, but also to general care that affirms and respects TGD patients. Gender-affirming care included supporting TGD patients in who they are in terms of affirming their gender identities and expressions, identifying and flexibly responding to TGD patient needs, creating an affirming environment and interactions, offering safe and welcoming spaces, and delivering care using principles that address systemic inequities TGD patients often experience in healthcare settings.

While not explicitly stated by our participants, concerns spanned across the four levels comprising the healthcare system [44, 45]: (1) Patient, (2) Care team, (3) Organization/institution (e.g., hospitals/clinics), and (4) Socio-political and economic environment [45, 46]. Given the interplay between levels, our findings suggest that healthcare providers have multiple opportunities to expand how they conceptualize and deliver gender-affirming healthcare, develop care networks, and advocate for systemic change. Our results also highlight the need to engage multiple key stakeholders to intervene structurally, including healthcare agencies, administrators and executives, health insurers, and policy makers to change policies and practices. This is especially true in the current socio-political environment, with increasing anti-trans legislation and laws limiting or banning gender-affirming care for minors and some young adults [47], which has created a climate of fear and avoidance of healthcare for TGD people.

Based on participant experiences, we developed the AFFIRM Framework, a practical and actionable tool for clinical settings and systems to guide delivery of responsive gender-affirming care for TGD adults. In Table 3 we offer actionable items and evaluation measures for each aspect of AFFIRM that providers and healthcare systems can use to optimize care for TGD patients. In the context of myriad physical and mental health inequities burdening TGD populations [2, 48], and the legacy of experiences of stigma and discrimination in healthcare settings [15], access to trauma-informed [49] gender-affirming care represents a vital intervention point to address interpersonal and structural barriers TGD people face when engaging in care systems. The authors acknowledge the difficulty of implementing many of these action items in the face of multiple points of resistance to change throughout healthcare system [46]. Yet, continued efforts are needed to advocate for healthcare system change, including addressing bureaucratic and systemic barriers that make navigating current health systems challenging for TGD patients and communities.

**Table 3:** AFFIRM Framework: Actionable steps for gender-affirming care systems and providers

Affirms patients in individual interactions	Uses chosen name, pronouns, and other gender-affirming language	1. Create intake forms that include space for individuals to enter chosen pronouns and name 2. Offer multiple ways for patients to provide chosen name, pronouns, and other identifiers to improve comfort (e.g., utilizing patient portals, online forms, verbal in office, etc.) 3. Use gender neutral language at the front desk and other public areas as to not “out” patients to other patients 4. Double check gender identity with the patient directly as their chart may not be up to date or they may not be comfortable having gender information in their chart 5. Utilize the language provided by the patient in verbal interactions and patient documentation (if permissible by your patient) 6. Use trauma-informed language throughout intake form—warn of potentially sensitive/triggering responses 7. Work with hospital, clinic, and insurance representatives to change policy around deadname use when it is still a legal name 8. Remove gendered signage throughout clinic spaces
	Has other TGD people present	1. Hire TGD staff members and allied health professionals, especially in TGD-specific health spaces 2. Hire TGD peer health navigators 3. Create affirming physical spaces to increase TGD patients’ uptake of services and presence in healthcare settings 4. Train TGD providers and create mentorship pipelines to increase the number of TGD providers 5. Continue to train cisgender providers in effective allyship and provision of TGD clinical care
	Eliminates transphobia from and inappropriate verbal and nonverbal interactions with providers	1. Be aware of body language when working with TGD patients 2. Avoid making assumptions about TGD people, desires for medical gender affirmation, sexual behaviors and activity, and healthcare needs by allowing patients to lead their visits 3. If you do need to ask a possibly sensitive question to a patient, frame why it is important for you to ask by describing how their answer can impact their care and respect patient’s decision to not respond to your question if they are not comfortable 4. Avoid stigmatizing statements about TGD people or celebrities 5. Participate in trainings delivered by TGD people, compensate TGD people for providing trainings, and engage in independent self-directed education to learn how to identify stigmatizing language and build tools to intervene in other provider’s problematic statements
	Recognizes TGD patients as experts and centers goal-oriented gender-affirming care	1. Utilize narrative-based approaches when interacting with patients as to center their experiences in the consult or appointment 2. Use patient-centered care to develop unique and individualized care plans for gender affirmation 3. Recognize that many patients have read and educated themselves about TGD health care and research and encourage providers to use office time to learn about TGD healthcare without placing the burden on patients 4. Consider the knowledge set patients bring to appointments based on their own research and spend time exploring that with them 5. Collaborate and allow the patient to “drive” their gender affirmation journey; avoid making assumptions about gender-affirming care desires (e.g., hormones, surgery) 6. Approach gender-affirming care from a harm reduction lens – an alive patient with a slightly higher health-related risk profile is preferable to a patient prematurely taking their own life due to lack of access to needed gender-affirming care)
Flexible and accessible	Makes gender-affirming care available beyond urban population-specific clinics, offers care at non-traditional times and settings, and addresses challenges with proximity to care	1. If applicable, use hospital satellite locations for some clinics to provide care to patients across a wide geographic area 2. Implement telehealth services 3. Advocate for cross-state licensure for telehealth 4. Troubleshoot parking and transportation barriers (for example, host clinics in spaces that are close to public transit) 5. Provide resource lists for local accommodations for patients who have extensive travel to access gender-affirming care 6. Hold non-traditional clinic hours for people who work weekdays 9a-5p
	Reduces wait times through facilitated linkages to gender-affirming providers	1. Expand the number of available gender-affirming providers by training medical students, residents, fellows, and allied health professional students in gender-affirming care 2. Train existing providers and teams in trauma-informed, gender-affirming care 3. Be intentional about “LGBTQ + friendly” labels on hospital, clinic, and provider websites (only list as LGBTQ + friendly if provider/setting is truly competent in working with TGD patients) 4. Develop a process to “vet” referrals to ensure providers are gender-affirming and keep a list of affirming providers on hand
	Increases disability-related Accessibility for TGD patients with disabilities	1. Ensure all spaces are physically accessible (e.g., ramps, working automatic door openers, etc.) 2. Consider using non-fluorescent lighting and white noise machines to mitigate potential sensory overload 3. Replace ableist imagery/signage throughout patient clinic space to be inclusive of TGD people with disabilities
	Increases financial accessibility of gender-affirming services	1. Support local and national mutual aid funds and share financial support information with patients 2. Support patients in insurance and employer advocacy 3. Increase the insurances accepted in healthcare clinics and practices for care reimbursement 4. Encourage patients to contact the organization’s patient billing solutions team for potential payment support options 5. Offer connection to case managers/staff if patients are facing financial difficulty and/or hire staff for support if case managers are not currently available
Fights systemic oppression	Dismantles gatekeeping practices related to gender-affirming care	1. Adopt informed consent models to eliminate gatekeeping practices. When working with insurance companies for coverage, encourage them to adopt the most recent standards of care from WPATH and the Endocrine Society to increase informed consent-based care coverage 2. Increase recruitment of a diverse clinical workforce 3. Assess all educational materials for readability, clarity, and transparency
	Understands intersectional experiences of TGD patients (e.g., racism, cisgenderism)	1. In addition to hiring TGD staff, actively hire staff of all backgrounds and intersectional experiences to ensure inclusivity 2. Train staff to prevent, identify, and combat microaggressions 3. Create systems of accountability and policies to address staff who do not change harmful actions
	Navigates complex power dynamics and medical culture to ensure TGD patient-centeredness	1. Consider use of an outside consultant to review office/staff culture and provide recommendations for addressing concerns 2. Encourage leadership to attend Community Advisory Board meetings (see text in next section) to receive feedback from patients and enhance communication with providers, clinical staff, and administrators
	Creates TGD Community Advisory Boards (CAB)	1. Form a CAB of local TGD community leaders and current patients to facilitate community trust and identify places for additional growth for larger or hospital-based TGD health programs 2. Encourage other local health facilities to form a CAB 3. Consider creating a city-wide or county-wide CAB to ensure clinics who are not likely to form a CAB themselves are still able to implement programs to meet TGD community-identified needs 4. Pay CAB members for their time and expertise
	Shows up to be present and sponsors TGD community events	1. Reach out to local community organizations to see how to best support local community efforts 2. Allow local community organizations and support groups to use clinic space as a “third space,” meaning a community space that is not the home or the workplace 3. Connect with other local health facilities, colleges, and shelters 4. Engage TGD communities in events that are not strictly health focused to facilitate trust and demonstrate a commitment to TGD communities beyond physical or mental health
	Addresses holistic gender-affirming care needs of TGD communities (ensures positive “word of mouth” communication in TGD communities)	1. Utilize trauma-informed care principles and methods in providing gender-affirming care 2. Establish reputation as gender-affirming in the community so “word of mouth” facilitates trust 3. Provide referrals to providers who are gender-affirming and competent in caring for TGD patients to meet the holistic needs of TGD communities
Retains patients in care	Recognizes the need for trauma-informed gender-affirming care	1. See clinical spaces as a natural opportunity to facilitate connections between TGD patients and trusted social work or mental health care providers 2. Continue to train and subsidize education costs for TGD mental health professionals 3. Hold space for TGD patients who are enduring harm outside of their physical health and understand how harm, discrimination, and abuse (experienced as trauma) have downstream impacts on physical health 4. Hold other providers accountable for abuse and discrimination
	Builds and rebuilds trust with TGD communities	1. Bridge the gap between pediatric and adult care with providers that are gender-affirming 2. Engage directly with TGD communities in ethical and appropriate ways outside of clinical settings 3. Financially support TGD communities, indicating direct commitment to the well-being of TGD patients beyond clinical care 4. Directly acknowledge the harms other providers may have caused in the past and directly share ways that current care provision will be supportive and not replicate past negative experiences 5. Create outreach programs for those TGD patients who have delayed or avoided care due to past or anticipated discrimination
	Provides TGD patients with gender-affirming care referrals for unmet needs	1. Create a list of providers for gender-affirming referrals, including specialties not related to gender-affirming care (e.g., hematology/oncology, orthopedics) 2. Regularly attend TGD health professional events to build local and national networks of gender-affirming providers 3. Utilize existing networks, such as the GMLA network (Health Professionals Advancing LGTBQ Equality), to identify local providers. Encourage gender-affirming providers to register themselves on GMLA and other listservs so they can easily be found by TGD patients and other providers
Multidisciplinary	Builds integrated and connected care teams, incorporates non-medical professionals (e.g., peer navigators), addresses broader TG health-related (e.g., social services)	1. Deliver care using a multidisciplinary team-based approach 2. Integrate delivery of multiple healthcare services (e.g., medical, mental and behavioral health, specialist care) 3. Hire social workers, peer advocates/patient navigators, and other support service providers who have expertise in navigating TGD-specific challenges and barriers (e.g., insurance, housing, legal document changes) 4. Encourage staff and providers to engage in continued education in the field or to attend organized events to enhance knowledge and competencies

## Limitations

This sample was geographically restricted to the Boston, Massachusetts area and was largely White and young (average age 28.5). This study may not be representative of those in gender-affirming care deserts, older populations, or a more racially diverse sample. Interviews were conducted in summer 2021, after the onset of the COVID-19 pandemic but prior to the fall 2022 influx of anti-transgender legislation across the U.S. [47]. Though Massachusetts has not proposed anti-transgender legislation, participants’ healthcare experiences and well-being may have been impacted by the geopolitical environment during which interviews were conducted. A strength of this study was having a primarily TGD analytic team as well as a TGD study PI, supported by cisgender clinical experts, offering a unique blend of lived and professional experiences throughout analysis and interpretation of findings.

## Conclusions

There is an urgent need for competent gender-affirming care for TGD people. Study findings and the novel AFFIRM Framework which emerged from patient narratives can be applied to optimize current gender-affirming care, inform future delivery of care, and set benchmarks for healthcare systems to provide high-quality care for this health disparities population. Further, the current study offers rare qualitative data to support what policy-making bodies such as Association of American Medical Colleges (AAMC), Lambda Legal, National Center for Transgender Equality (NCTE), Advocates for Trans Equality (A4TE), and other leading organizations have put out as recommendations for healthcare systems to make TGD care more accessible. Findings can be used to advance these recommendations and enhance provision and access to gender-affirming care for TGD people in the U.S.

## Supplementary Information

Supplementary Material 1.

## Data Availability

Data cannot be shared publicly because interview transcripts have sensitive data (e.g., extensive health histories) that could be used to harm or identify participants by their health providers. Researchers may contact the corresponding author to determine whether they meet the criteria to access confidential data and discuss potential project ideas.

## Abbreviation

TGD: Transgender, Nonbinary, and Gender Diverse
