# Supplementary material for: The AFFIRM Framework for gender-affirming care: qualitative findings from the Transgender and Gender Diverse Health Equity Study
Source: BMC Public Health. 2025 Feb 6;25:491. doi: 10.1186/s12889-024-21261-7 (PMC11800641; doi:10.1186/s12889-024-21261-7)
Supplement: Supplementary file 1 — Supplementary Material 1. [file 12889_2024_21261_MOESM1_ESM.docx]

Supplementary Materials

Table 1. Semi-structured qualitative interview guide: Transgender and Gender Diverse (TGD) Health Equity Study.

| **Interview Domain** | **Semi-Structured Questions** |
| --- | --- |
| TGD Care Experiences | 1. What kinds of care do you receive that are related to your experience as a transgender or gender diverse individual? 2. Where do you receive this care (i.e., large hospital system, community health center, private practice, LGBTQ+ specific care facility)? 3. Have you had any notably good experiences with your provider(s)? If yes, please describe the experiences. 4. Have you had any notably bad experiences with your provider(s)? If yes, please describe the experiences. (probe: What do you wish went differently?) 5. Have you experienced wait times or been on a wait list for any TGD care (i.e., mental health services or surgical services)? (probes: Was this wait time acceptable? What are acceptable wait times?) |
| Considerations for TGD Care | 1. In your ideal world, what are the top 2-3 things you want your provider to know and understand about your experience as a transgender or gender diverse individual, particularly in the context of healthcare? 2. What training and education do you want your providers to have? What about other staff (ex. administrators, social workers, peer navigators, etc.) at health centers and hospitals? 3. Patients are sometimes asked about their GI (gender identity) and ASAB (assigned sex at birth) at their registration or during their visit. What are your experiences with this process? (probes: What would you do differently? Have you used a patient portal to update your information? When you provide this info is it used correctly by your care providers? Has there ever been an error with your gender identity that affected your care?) 4. Is there anything else you would like to share at this time? |

Table 2. Brief demographic questions: Transgender and Gender Diverse (TGD) Health Equity Study.

| **Variable** | **Response Options** |
| --- | --- |
| Age in Years |  |
| Gender identity |  |
|  | Transgender man |
|  | Transgender woman |
|  | Nonbinary |
|  | Genderqueer |
|  | Agender |
|  | Did not specify |
| Sex assigned at birth |  |
|  | Female |
|  | Male |
|  | Choose not to answer |
| Race/ethnicity |  |
|  | Asian |
|  | Black/African American |
|  | White |
|  | Hispanic/Latino |
|  | Multiracial |
|  | Choose not to answer |
| Insurance Status |  |
|  | Medicaid or MassHealth |
|  | Medicare |
|  | Private insurance |
|  | Other, did not specify |
| Education |  |
|  | Completed high school |
|  | Some college or associates degree |
|  | Completed 4-year college/bachelor’s degree |
|  | Some graduate school |
|  | Completed graduate school |
| Attempted to receive transition/gender affirmation related care in the past 6 months |  |
|  | Yes |
|  | No |
|  | Choose not to answer |
